# Comparisons of angular momentum at takeoff in six types of jumps in women's figure skating

**DOI:** 10.3389/fspor.2025.1597598

**Published:** 2025-08-29

**Authors:** Mizuki Yamaguchi, Shinji Sakurai

**Affiliations:** ^1^ Teacher, Librarian and Curator Education Center, Aichi Shukutoku University, Nagakute, Japan; ^2^ School of Health and Sport Sciences, Chukyo University, Toyota, Japan

**Keywords:** figure skating, jump, takeoff, angular momentum, motion analysis

## Abstract

**Introduction:**

In figure skating, various types of multirotational jumps are becoming increasingly important. Based on mechanical considerations, the angular momentum of the entire body at takeoff can increase the number of rotations in the air. This study aimed to compare and characterize the angular momentum of the entire body and each body part during six different jumps.

**Methods:**

Seven female figure skaters performed six double jumps on the ice. The positions of the markers, which were attached to anatomical landmarks, were recorded using a three-dimensional motion analysis system. The angular momenta of the entire body and each body part (trunk, arms, and legs) at takeoff were calculated. The angular momentum was further divided into the transfer term generated by the motion of the body part around the body's center of mass and a local term generated by the rotational motion of the body part itself. A paired t-test was performed to compare all jumps, and multiple comparisons were performed using Holm's method.

**Results and discussion:**

The angular momentum of the entire body at takeoff was similar for all jumps. Although the trunk generated a large local term that was similar in all the jumps, the arms and free leg generated large transfer terms with different patterns. This suggests that different strategies may be used to generate angular momentum at takeoff depending on the jump type.

## 1 Introduction

In figure skating, multirotational jumps are becoming increasingly important. It involves the following six types of jumps, in order of increasing difficulty: axel, Lutz, flip, loop, Salchow, and toe loop (Table 1). Jumps with a higher difficulty and more rotations have higher base values (1). The final score for a jump is determined by adding the base value to the quality and shortage of jump rotations. In competitions, skaters must perform multiple types of jumps according to rules. A study analyzing the number of jumps performed in singles events at the World and European Championships from 2005 to 2019 reported that the number of double jumps performed by the top five skaters at each competition decreased for both men and women, whereas the number of triple and quadruple jumps increased (2). In 2022, the quadruple axel was successfully performed for the first time in a competition in men's single skating (3). Quadruple jumps, excluding the axel, have been successfully performed in women's competitions. Skaters must perform various types of multirotational jumps to achieve good results in competitions.

**Table 1:** Jump types and methods with counterclockwise rotations.

Jump	Base value			Approach direction	Supporting leg on approach	Takeoff leg or toe-pick leg	Free leg
	2 rev.	3 rev.	4 rev.				
Edge jump							
Axel	3.30	8.00	12.50	Forward	Left	Left	Right
Loop	1.70	4.90	10.50	Backward	Right	Right	Left
Salchow	1.30	4.30	9.70	Backward	Left	Left	Right
Toe jump							
Lutz	2.10	5.90	11.50	Backward	Left (outside)	Right	Left
Flip	1.80	5.30	11.00	Backward	Left (inside)	Right	Left
Toe loop	1.30	4.20	9.50	Backward	Right	Left	Right

Takeoff legs are used in edge jumps and toe-pick legs are used in toe jumps.

Table 1 shows jump methods with counterclockwise rotations, which many skaters perform. For example, in the axel, the skater takes a forward approach with the left leg (supporting leg), swings the right leg up (free leg), and jumps with the left leg (takeoff leg). In the Lutz, the skater takes a backward approach with the left leg, swings the toe of the right leg (toe-pick leg) down onto the ice surface (this motion is a “toe pick”), and then jumps with the outside edge of the left leg. At this point, the supporting leg leaves the ice surface before the toe-pick leg and swings up to become the free leg. The toe-pick leg then leaves the ice surface. The axel, loop, and Salchow, which are jumps where the free leg swings up, are called “edge jumps.” In contrast, the Lutz, flip, and toe loop, which are jumps where the toe pick is used, are called “toe jumps.” All jumps involve landing with the right leg, facing backward. Only the axel, which uses a forward approach, requires an extra half rotation in the air compared with the other jumps.

To successfully perform a multirotational jump, the body must be rotated to the required extent in air. A previous study reported that in the Olympic women's single free skating program, the higher the number of rotations in jumps, the greater the jump height. However, it also reported that the jump height of a single Lutz was greater than that of a double Lutz; therefore, the possible number of rotations in the air was not necessarily determined by the jump height alone (4). In contrast, another study, analyzing single, double, and triple axels of male Olympic athletes, reported that all jumps had similar jump heights and vertical velocities at takeoff, whereas the rotational velocity increased with the increasing number of rotations (5). Furthermore, it was reported a greater rotational velocity in the air for the men's quadruple toe loop than for the triple toe loop (6). These studies suggest that in multirotational jumps, an increase in the rotational velocity at takeoff is important.

The rotational velocity of the body in the air is related to the angular momentum of the entire body at takeoff based on mechanical considerations. In the air, rotational motion occurs around the body's center of mass (COM). At this time, the angular momentum of the entire body is the product of the moment of inertia around the axis passing through the body's COM and the angular velocity (rotational velocity). Assuming that air drag is negligible, there is no external torque in the air, thus angular momentum is conserved and constant. The moment of inertia can be reduced by pulling the upper and lower limbs toward the trunk. Therefore, generating a large angular momentum at takeoff and reducing the moment of inertia in the air increases the rotational velocity in the air, thereby increasing the number of rotations. Thus, skaters must generate a sufficiently large angular momentum at takeoff.

A few studies have been conducted on the angular momentum at takeoff in figure skating. A previous study, analyzing single and double axels in men and women, reported that a large angular momentum was generated by the free leg during the approach (7). Another study reported that in the double axel, the angular momentum of the upper limb with weighted gloves increased compared with that without gloves (8). Research on high jump, which involves approach running, take-off movement, and rotating in the air, similar to figure skating jump, may also be useful for understanding the relationship between angular momentum and rotational motion.

In a study of the Fosbury-flop, it was reported that most of the body's angular momentum was generated during the takeoff phase. Furthermore, most of the vertical component of angular momentum was attributed to the lead leg (the opposite leg of takeoff leg). The movements of both arms were indicated to contribute positively or negatively to the vertical angular momentum, depending on the jumping method (9). In figure skating jumps, although the primary rotation axis differs from high jump (vertical vs. horizontal), the angular momentum could be generated by swinging the arms and legs just before takeoff for rotation in the air, similar to high jump.

The studies mentioned above (7, 8) did not show the angular momentum of the entire body or the body parts other than the ones focused on. In addition, few studies have examined the angular momentum of jumps other than the axel. There is still insufficient foundational knowledge to discuss the role of angular momentum in rotational movements and to clarify the mechanism for six types of jumps in figure skating. With the goal of addressing this issue in the future, we first focused on angular momentum at takeoff in this study.

The purpose of this study was to compare and characterize the angular momentum of the entire body and each body part at takeoff for six types of figure skating jumps. The hypotheses of this study were as follows: (1) the axel, which has an extra half rotation compared to the other jumps, would have the largest amount of angular momentum in the entire body, while the other jumps would have the same amount, and (2) the angular momentum of each body part would differ among jumps because of the different jumping methods.

## 2 Materials and methods

### 2.1 Participants

Seven Japanese female skaters (age: 20.0 ± 1.4 years; height: 1.55 ± 0.04 m; body mass: 46.9 ± 3.7 kg; competition history: 12.4 ± 2.0 years) participated in this study. All were able to perform six types of double jumps and at least two types of triple jumps and had a skill level sufficient to compete in the Japanese National Championships. This study focused on double jumps that all participants could successfully perform. The direction of rotation was counterclockwise for all participants. Informed consent was obtained from all participants (or their guardians, if minors) before starting the experiment. This study was approved by the Ethics Committee of Chukyo University (approval number: 2020-17).

### 2.2 Data collection

**Figure 1:**
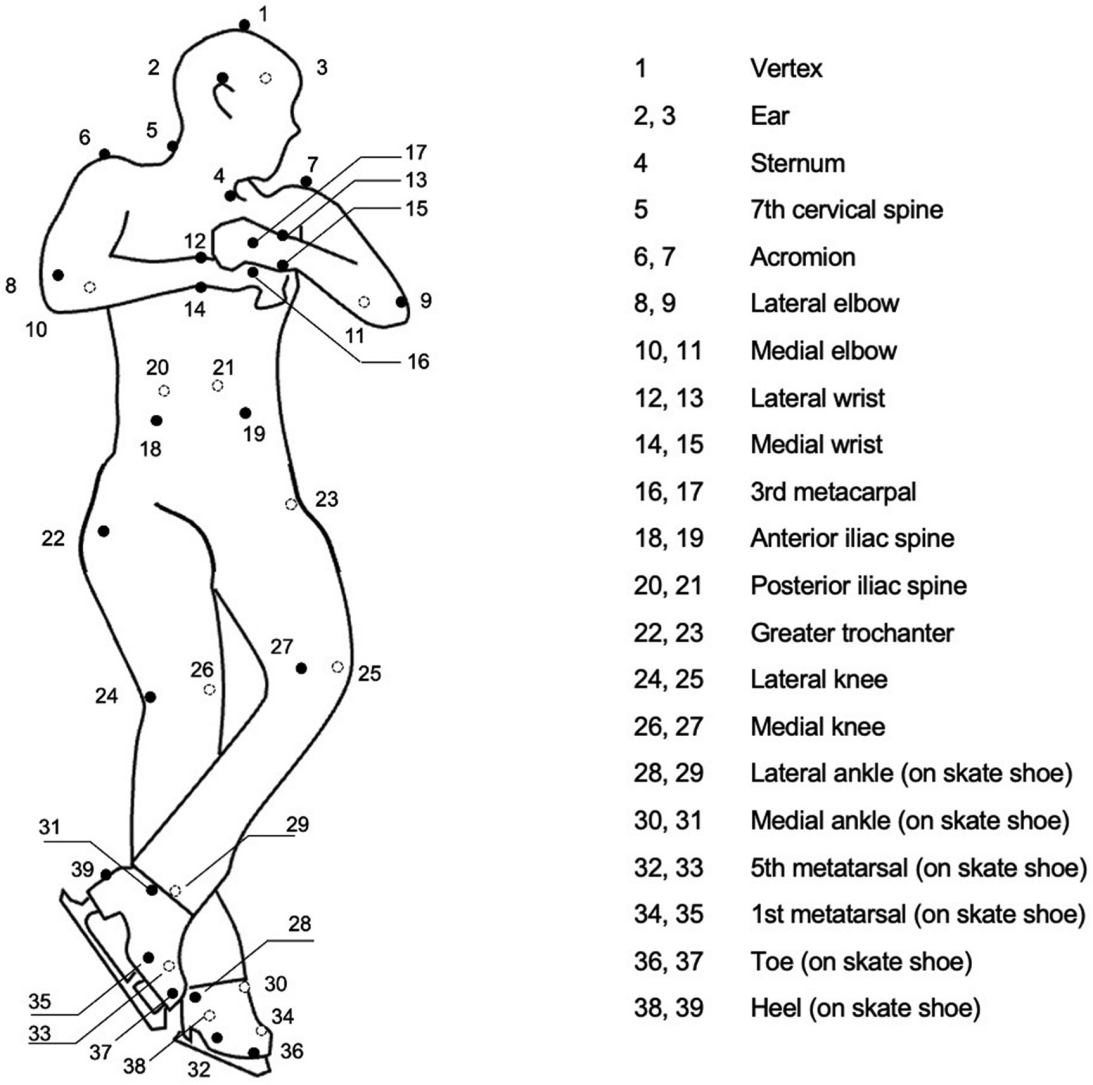
Placement of reflective markers.

Six types of double jumps were performed in an order determined freely by the participants in the ice skating rink at Chukyo University, Japan. Participants were instructed to perform a successful double jump in each trial. Hence, they made greater efforts with jumps they were not good at. The participants wore compression clothing and skates. Retroreflective markers were attached to 39 anatomical landmarks on the participant's clothing and skates (Figure 1). Marker positions during jumps were recorded using a three-dimensional motion analysis system (Vicon MX and T-20S, Vicon Motion System, UK). A high-speed digital video camera (Fastec, Fastec Imaging) was also used to determine the takeoff and touchdown. The sampling frequency was 500 Hz for both the motion analysis system and high-speed camera, which were electrically synchronized. For each jump, the jump judged to be successful by another skilled figure skater was analyzed.

### 2.3 Data analysis

**Figure 2:**
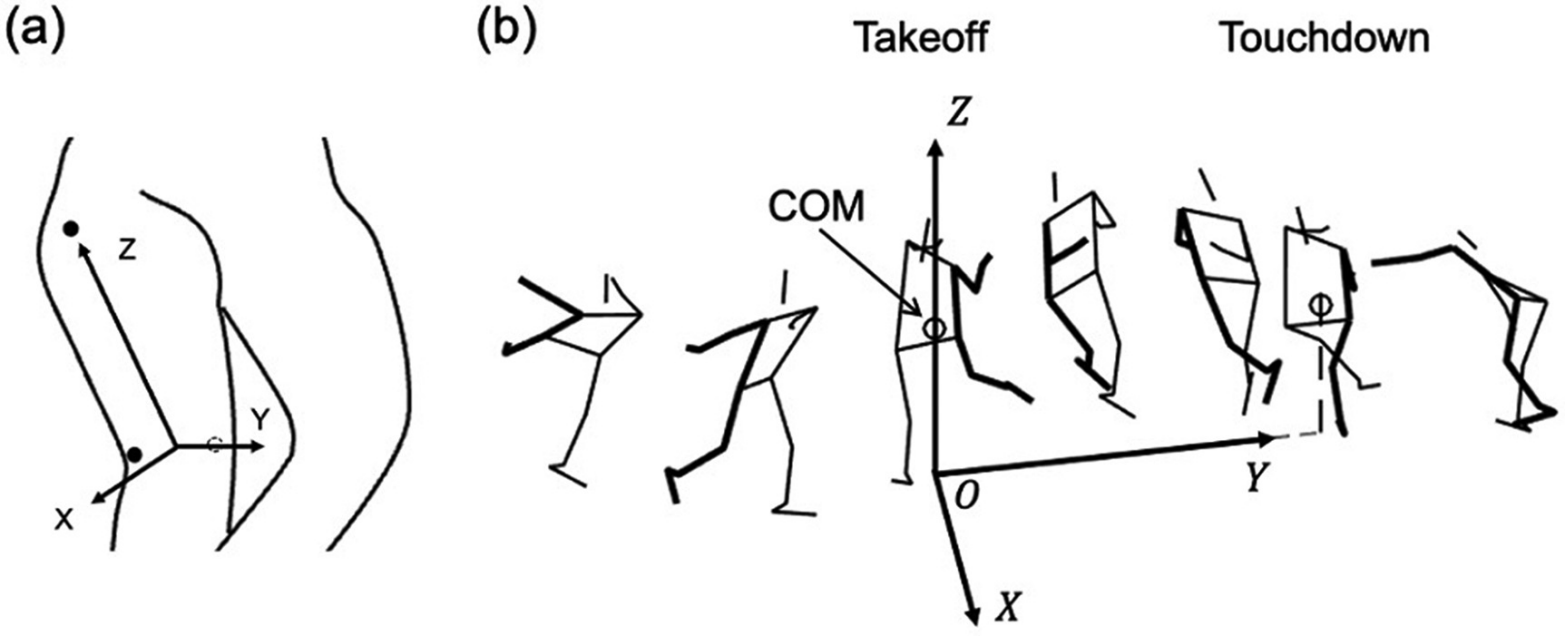
**(a)** The local coordinate system of body segment (showing only right thigh). The Z-axis of the segment was set to its longitudinal direction, the *Y*-axis was set to its posterior-to-anterior direction, and the *X*-axis was set to its left-to-right direction perpendicular to Y- and Z-axes. **(b)** The global coordinate system. The origin was the position of the body's center of mass (COM) on the horizontal plane (ice surface) at the takeoff. The *Y*-axis was set to the COM displacement on the horizontal plane from takeoff to touchdown. The Z-axis was the vertical direction. The *X*-axis was perpendicular to the Y- and Z-axes.

The collected marker position coordinates were smoothed using a fourth-order low-pass Butterworth filter at a cutoff frequency of 12 Hz. A rigid body-linked segment model consisting of 14 segments (head, torso, and right and left upper arms, forearms, hands, thighs, lower legs, and feet) was constructed to calculate the COM position. The inertia properties of the body were applied, as previously described (10). The local coordinate system (LCS) of a segment was defined as the Z-axis in the longitudinal direction, *Y*-axis in the posterior-to-anterior direction, and *X*-axis in the left-to-right direction (Figure 2a). The mass of the skates was set to 1 kg per side for all participants. The centers of mass of the shoe and foot were assumed to coincide and were considered together as the foot segment. Therefore, the participants' total mass was 2 kg greater than their body mass.

The global coordinate system (GCS) was defined (Figure 2b). The *Y*-axis was defined as the COM displacement from takeoff to touchdown in the horizontal plane. The Z-axis was defined as the vertical direction, and the *X*-axis was defined as perpendicular to the Y- and Z-axes in the right-hand coordinate system. Finally, the COM position on the horizontal plane at takeoff was defined as the origin.

The basic jump parameters were calculated, as follows. The flight time was determined by visually checking the takeoff and touchdown from the movie recorded by a high-speed camera. Takeoff was defined as the first frame in which the skate blade left the ice surface, whereas touchdown was defined as the first frame in which the skate blade contacted the ice surface. The rotational angle was defined as the angle at which the shoulders rotated in the air. The rotational velocity was determined by dividing the rotational angle by the flight time.

### 2.4 Calculation of angular momentum

The angular momentum of the entire body and each body part was calculated for all data points, and the values at takeoff were statistically analyzed (shown in the following section). The angular momentum of the i^th^ segment ($L_{i}$) was calculated separately from the transfer ($L_{i}^{transfer}$) and local ($L_{i}^{local}$) terms, as follows:

$$\begin{array}{c} L_{i}=L_{i}^{transfer}+L_{i}^{local}. \end{array}$$

The transfer term was the angular momentum generated by the motion of the segment around the COM and was calculated as follows:

$$\begin{array}{c} L_{i}^{transfer}=r_{i}\times m_{i}v_{i}, \end{array}$$

where $m_{i}$ is the segment mass estimated from previous study (10), $r_{i}$ is the position vector of the segment center of mass relative to the COM, and $v_{i}$ is the velocity vector of the segment center of mass relative to the COM.

In contrast, the local term was the angular momentum generated by the rotational motion of the segment itself, which was calculated as follows:

$$\begin{array}{c} L_{i}^{local}=R_{i}I_{i}^{\prime }\omega_{i}^{\prime }, \end{array}$$

$$\begin{array}{c} R_{i}=\left(\begin{array}{ccc} i_{i} & j_{i} & k_{i} \end{array}\right), \end{array}$$

$$\begin{array}{c} \omega_{i}^{\prime }={\left(\begin{array}{ccc} k_{i}\cdot \frac{dj_{i}}{dt} & i_{i}\cdot \frac{dk_{i}}{dt} & j_{i}\cdot \frac{di_{i}}{dt} \end{array}\right)}^{T}, \end{array}$$

$$\begin{array}{c} I_{i}^{\prime }=\left(\begin{array}{ccc} I_{i,xx}^{\prime } & 0 & 0\\ 0 & I_{i,yy}^{\prime } & 0\\ 0 & 0 & I_{i,zz}^{\prime } \end{array}\right), \end{array}$$

where $R_{i}$ is the coordinate transformation matrix from the i^th^ LCS to the GCS; $\omega_{i}^{\prime }$ is the angular velocity vector of the segment expressed in the LCS; $I_{i}^{\prime }$ is the inertia moment tensor around the segment's principal axis of inertia expressed in the LCS; $i_{i}$, $j_{i}$, and $k_{i}$ are the unit vectors in each axis of the i^th^ LCS; and $I_{i,xx}^{\prime }$, $I_{i,yy}^{\prime }$, and $I_{i,zz}^{\prime }$ are the principal moments of inertia dependent on the length and mass of the segment. The LCS was assumed to coincide with the principal axes of inertia (10).

The angular momentum of each body part was divided into five parts: trunk (head and torso), right and left arms (upper arm, forearm, and hand), and right and left legs (thigh, lower leg, and foot). The right and left legs were categorized as the takeoff, toe-pick, and free legs, respectively (Table 1). The angular momentum of the entire body was obtained by summing the angular momentum of each body part. The sum of the transfer and local terms was also obtained. All angular momenta were normalized by dividing by the product of the participant's total body mass and height squared (11). The ratio of the angular momentum of each body part to that of the entire body was also calculated.

In figure skating jumps, the rotational motion around the vertical axis is the most important. Therefore, this study analyzed only the Z components of the calculated angular momentum vectors.

### 2.5 Statistical analysis

Prior to statistical analysis, the Shapiro–Wilk test was performed to examine the normality of each variable using by IBM SPSS Statistics (version 30.0; IBM Corp.). As a result, one jump was found to be non-normal for each of the five angular momentum variables. It was unlikely that only one of the six jumps would be non-normal for the same variable. Therefore, in this study, all variables were assumed to follow a normal distribution.

For the variables obtained, paired t-tests were applied to all jump combinations, and multiple comparisons were performed using Holm's method (12). The adjusted probability of significance was set at *p* < 0.05. MATLAB software (version R2024a; Mathworks Inc.) was used for the data and statistical analyses.

## 3 Results

### 3.1 Basic jump parameters

Table 2 lists the basic jump parameters. For the flight time, comparisons of six different jumps showed significant differences between the axel and Salchow from the others (*p* = 0.004–0.039; all p-values were adjusted by Holm's method). For the rotational angle, comparisons of five different jumps, except for the axel, showed no significant differences between all jumps (*p* > 0.677). For the rotational velocity, comparisons of five different jumps, except for the axel, showed significant differences between the Salchow and the others (*p* = 0.005–0.049).

**Table 2:** Basic jump parameters.

Jump (symbol)	Flight time (s)		Rotational angle (deg)		Rotational velocity (deg s^−1^)	
Edge jump						
Axel (A)	0.493 ± 0.030	L, ***S***	700.6 ± 34.0	–	1422.6 ± 61.8	–
Loop (L)	0.443 ± 0.025	A	470.5 ± 23.9		1063.9 ± 54.7	
Salchow (S)	0.419 ± 0.014	***A*** , Lz, F, ***T***	463.4 ± 25.6		1108.9 ± 83.6	Lz, ***T***
Toe jump						
Lutz (Lz)	0.481 ± 0.043	S	487.0 ± 31.7		1015.8 ± 41.8	S
Flip (F)	0.479 ± 0.043	S	489.2 ± 30.4		1024.8 ± 48.6	
Toe loop (T)	0.471 ± 0.028	***S***	476.7 ± 27.7		1013.4 ± 61.5	***S***

Values are shown as mean ± SD. Significant differences from other jumps are indicated with jump symbols when Holm's adjusted *p* < 0.05, with bold-italic symbols when *p* < 0.01; for example, flight time of the axel jump significantly differed from the loop (*p* < 0.05) and Salchow (*p* < 0.01). Six different jumps were compared for flight time. Five different jumps, except for the axel (shown as the symbol “–”), were compared for rotational angle and rotational velocity.

### 3.2 Angular momentum of the entire body

**Figure 3:**
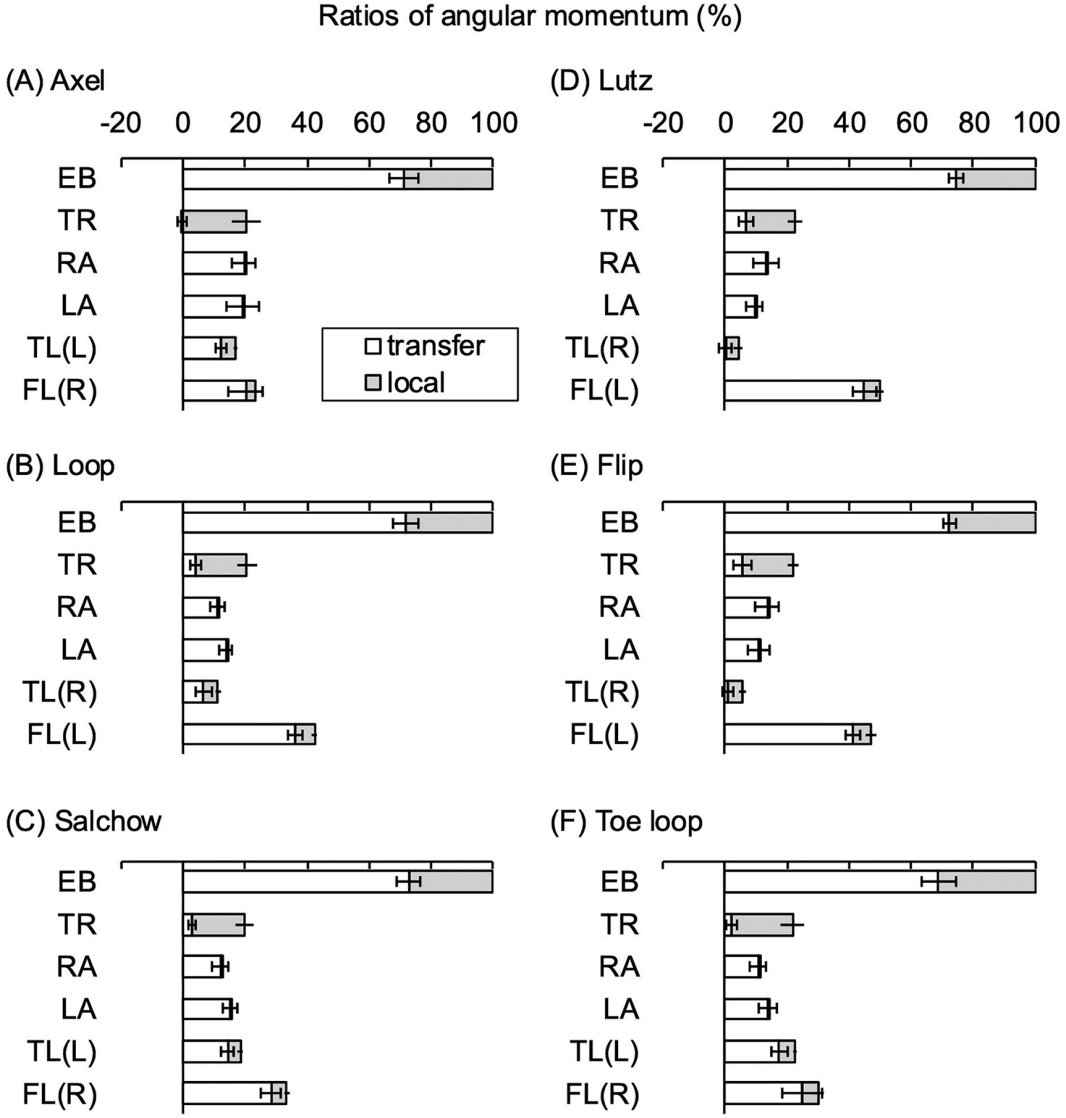
Ratios of the angular momentum of each body part to that of the entire body for six types of jumps; **(A)** axel, **(B)** loop, **(C)** Salchow, **(D)** Lutz, **(E)** flip and **(F)** toe loop. Standard deviations are shown as horizontal bars with caps for transfer terms and without caps for local terms. EB, entire body; TR, trunk; RA, right arm; LA, left arm; TL, takeoff leg for axel, loop, and Salchow, and toe-pick leg for Lutz, flip and toe loop; FL, free leg; R or L, right or left side of leg.

Table 3 lists the normalized angular momenta of the entire body and each body part around the vertical axis through the COM at takeoff. The angular momentum of the entire body was the sum of the angular momenta of the trunk, right and left arms, takeoff or toe-pick leg, and free leg. The transfer and local terms are also shown. Figure 3 shows the ratio of the angular momentum of each body part to that of the entire body.

**Table 3:** Angular momenta of entire body and each body part around the vertical axis through the body's center of mass at takeoff.

(×10^−3^ s^−1^)	Edge jump						Toe jump					
	Axel (A)		Loop (L)		Salchow (S)		Lutz (Lz)		Flip (F)		Toe loop (T)	
Entire body	152.1±7.1	F	140.2 ± 11.2		143.7 ± 7.1		138.7 ± 9.8		134.8 ± 10.7	A	133.4 ± 10.5	
transfer	108.6 ± 10.3		100.4 ± 12.0		104.3 ± 4.3		103.3 ± 9.0		97.7 ± 9.6		92.0 ± 10.6	
local	43.6 ± 6.5		39.7 ± 5.2		39.4 ± 6.4		35.4 ± 3.1		37.2 ± 2.3		41.4 ± 7.3	
Trunk	30.1 ± 6.1		28.9 ± 4.8		28.8 ± 6.1		31.4 ± 3.3		29.9 ± 5.2		28.9 ± 6.1	
transfer	−0.4 ± 2.0	Lz	5.9 ± 3.0		4.2 ± 2.0	Lz	9.7 ± 3.3	A, S, ***T***	7.6 ± 4.0		2.9 ± 2.3	***Lz***
local	30.6 ± 6.7		23.0 ± 3.8		24.6 ± 5.1		21.7 ± 3.0		22.3 ± 2.0		26.0 ± 5.3	
Right arm	30.7 ± 6.3	L, S, ***T***	16.6 ± 3.9	A	18.1 ± 3.4	A, T	18.8 ± 6.1		19.0 ± 5.7		14.9 ± 4.0	***A*** , S
transfer	29.8 ± 6.2	L, S, ***T***	15.7 ± 3.9	A	17.1 ± 3.5	A, T	18.1 ± 6.1		18.3 ± 5.7		14.2 ± 4.1	***A*** , S
local	0.9 ± 0.2		0.9 ± 0.2	***Lz*** , F	1.0 ± 0.2	***Lz*** , ***F***, T	0.7 ± 0.2	***L*** , ***S***	0.7 ± 0.2	L, ***S***	0.7 ± 0.2	S
Left arm	30.1 ± 7.9	Lz	19.7 ± 1.9		22.5 ± 3.4	F	13.6 ± 3.4	A	15.3 ± 4.1	S	19.0 ± 4.5	
transfer	29.4 ± 7.9		19.2 ± 1.9		21.8 ± 3.4		13.1 ± 3.5		14.8 ± 4.2		18.5 ± 4.5	
local	0.7 ± 0.2		0.5 ± 0.2		0.6 ± 0.1		0.5 ± 0.2		0.5 ± 0.2		0.5 ± 0.1	
	Left		Right		Left		Right		Right		Left	
Takeoff or toe-pick leg	25.7 ± 2.9	***L***	15.5 ± 3.2	***A*** , ***S***	26.4 ± 3.3	***L***	5.7 ± 3.9	***T***	7.3 ± 1.8	***T***	30.2 ± 4.3	***Lz*** , ***F***
transfer	18.8 ± 2.6	***L***	9.1 ± 3.6	***A*** , ***S***	20.5 ± 3.4	***L***	0.1 ± 3.1	***T***	1.2 ± 2.4	***T***	23.3 ± 4.1	***Lz*** , ***F***
local	6.9 ± 0.9		6.4 ± 1.1		5.9 ± 1.0		5.6 ± 2.0		6.1 ± 1.5		6.9 ± 0.8	
	Right		Left		Right		Left		Left		Right	
Free leg	35.5 ± 8.7	***L*** , S	59.6 ± 7.7	***A*** , S	47.9 ± 4.0	A, L	69.2 ± 8.2	***T***	63.3 ± 7.3	***T***	40.4 ± 6.3	***Lz*** , ***F***
transfer	31.0 ± 9.2	***L*** , S	50.5 ± 6.7	***A***	40.6 ± 3.8	A	62.4 ± 7.0	F, ***T***	55.8 ± 6.2	Lz, ***T***	33.1 ± 8.0	***Lz*** , ***F***
local	4.5 ± 0.8	***L*** , ***S***	9.1 ± 1.6	***A*** , S	7.3 ± 1.3	***A*** , L	6.8 ± 1.9		7.6 ± 2.5		7.4 ± 2.5	

Values are shown as mean ± SD, normalized by participant's mass and height. Significant differences from other jumps are indicated with jump symbols when Holm's adjusted *p* < 0.05, and with bold-italic symbols when *p* < 0.01. Legs were compared among edge jumps or toe jumps, and were indicated left or right side.

For the angular momentum of the entire body, comparisons of six different jumps showed a significant difference between the axel and flip (*p* = 0.043; *p* > 0.148 for the other comparisons). No significant differences in either transfer or local terms were found between jumps (*p* > 0.121).

### 3.3 Angular momentum of each body part

Comparisons of the six jumps showed no significant differences in the angular momentum of the trunk (*p* > 0.994). For the transfer term, the Lutz was significantly different from others (*p* = 0.005–0.021). No significant differences were found in local terms (*p* > 0.224).

Comparisons of six different jumps showed significant differences in the angular momentum of the right arm between axel and the other jumps and between the Salchow and toe loop (*p* = 0.008–0.038; *p* > 0.112 for the other comparisons). A similar trend was found for the transfer term (*p* = 0.009–0.046). Although some significant differences were found for the local term (*p* = 0.004–0.039), the magnitudes were <1.0 × 10^−3^ s^−1^.

Comparisons of the six different jumps showed only two significant differences in the angular momentum of the left arm between the axel and Lutz and between the Salchow and flip (*p* = 0.046 and *p* = 0.049, respectively; *p* > 0.050 for the other comparisons). No significant differences were found in transfer or local terms (*p* > 0.050). The magnitudes of the local terms were <0.7 × 10^−3^ s^−1^.

Comparisons between the three different edge or toe jumps showed significant differences in the angular momenta of the takeoff and toe-pick legs between jumps (*p* < 0.002; *p* > 0.246 for the other comparisons). A similar trend was observed for transfer terms (*p* < 0.002). No significant differences were found in either local term (*p* > 0.176).

Comparisons between the three different edge or toe jumps showed significant differences in the angular momenta of the free leg between jumps (*p* < 0.031; *p* = 0.051 for the other comparison). A similar trend was observed in the transfer terms for both the edge and toe jumps (*p* < 0.001). Significant differences in the local terms were found among all edge jumps (*p* < 0.042), whereas no significant differences were found among the toe jumps (*p* > 0.130).

## 4 Discussion

In this study, the angular momenta of the entire body and each body part were calculated in detail. In previous studies, Dapena's method (13) was used to calculate the angular momentum (7, 8). However, this method can result in large errors in the angular momentum because the products of inertia are ignored (14). We calculated the products of inertia for all segments of the body and presented more accurate angular momenta. Furthermore, by simultaneously comparing six different types of jumps, we clarified the angular momentum characteristics of each type of jump.

### 4.1 Angular momentum of the entire body

The angular momentum of the entire body in the axel was not significantly larger than that in the other jumps. The axel requires an extra half rotation in the air compared to the other jumps. The rotational angle, representing the actual number of rotations, and the rotational velocity of the axel were greater than those of the other jumps (Table 2). As mentioned in the introduction, angular momentum is the product of the moment of inertia and rotational velocity. Therefore, we predicted that in the axel, to achieve the required rotational velocity for two and a half rotations in the air, the body would generate a larger angular momentum than in the other jumps at takeoff (Hypothesis 1). However, no significant differences in either the transfer or local terms were observed between jumps; therefore, the angular momentum of the entire body had approximately the same magnitude (Table 3).

The rotational velocity in the air is related to the moment of inertia and flight time. Because the angular momentum of the entire body is constant in the air, decreases in the moment of inertia cause increases in the rotational velocity. In the axel, a large angular momentum of the entire body is generated at takeoff, and then the upper and lower limbs are drawn toward the trunk in the air to decrease the moment of inertia, thus causing a greater rotational velocity in the air. However, previous studies have indicated that the number of rotations in the air would be increased by the greater vertical velocity at takeoff, to obtain an increased flight time (6). If the rotational velocity in the air is small but the flight time is sufficiently long, the required number of rotations may be achieved. However, there is no consistent relationship between rotational and vertical velocities at takeoff (4, 5). In the present study, the flight time of the axel was not significantly greater than that of all other jumps. Moreover, despite having the shortest flight time, the Salchow tended to have a greater rotational velocity than the other four jumps, except for the axel (Table 2). The participants in this study likely achieved the required rotational velocity for two or two and a half rotations not only by generating angular momentum at takeoff but also by adjusting the moment of inertia in the air, rather than by increasing the flight time.

### 4.2 Angular momentum of each body part

To generate a large angular momentum for the entire body at takeoff, each part of the body must generate a large angular momentum. The six types of jumps have different directions and supporting legs during the approach and different takeoff, toe-pick, and free legs at takeoff (Table 1). Therefore, we predicted that even if the angular momentum of the entire body was the same, the angular momentum of each body part at takeoff would differ between jumps (Hypothesis 2). In this study, we compared and characterized the angular momentum of five body parts (the trunk, right and left arms, and right and left legs) between jumps.

#### 4.2.1 Angular momentum of the trunk

The angular momentum of the trunk was similar for all jumps. All jumps had a large proportion of local terms and a small proportion of transfer terms (Figure 3). The local term is the product of the moment of inertia of the segment and angular velocity. The moment of inertia depends on the segment mass. In addition, in the short time before the takeoff, a rotational movement of the body with the toe of the blade of the takeoff or toe-pick leg occurs (i.e., a “pivot”) (6, 7). In the present study, the body rotated by approximately 240° (200° in the axel) during takeoff (Table 2). Therefore, all jumps likely generate large local terms of angular momentum by rotating the high-mass trunk before takeoff.

In contrast, the transfer term depends on the relative position and velocity of the segment and COM. The inclination of the torso at takeoff is believed to increase the relative position of the COM on the horizontal plane, resulting in a larger transfer term. The tendency for the transfer term in the Lutz to be larger than that in the other jumps suggests an inclination of the torso at takeoff. The trunk and COM could also have the same degree of forward velocity at takeoff; therefore, the relative velocities of the two and the transfer term could be smaller.

#### 4.2.2 Angular momenta of arms

The angular momenta of the right and left arms were dominated by the transfer terms for all jumps, and local terms were negligibly small (Figure 3). The transfer terms for the right arm tended to be larger for the axel than for the other jumps. The transfer terms for the left arm showed no significant differences between jumps; however, the mean value for the axel was the largest and was similar in magnitude to that of the right arm (Table 3). In the axel, both arms quickly and simultaneously swing up from behind the body to the front of the body before takeoff. The ratio of the angular momentum of the free leg to the angular momentum of the entire body is smaller in the axel than in the other jumps, as discussed below (Figure 3). To compensate for this, the axel may involve both arms to generate greater angular momentum than the that in other jumps. In contrast, in jumps other than the axel, the right and left arms swing separately, following the body's rotational motion before takeoff. Thus, a slight difference between the right and left arms may have occurred (Figure 3).

#### 4.2.3 Angular momenta of legs

The angular momenta of the right and left legs were dominated by the transfer terms in all jumps, except for the toe-pick leg of the Lutz and flip, whereas the local terms were small (Figure 3). The angular momenta of both the takeoff and toe-pick legs were smaller than that of the free leg. Pushing on the ice surface with these legs at takeoff swings the free leg and generates vertical velocity in the body (5, 6). At this time, the takeoff and toe-pick legs are located below the COM, and their relative velocity to the COM is small, resulting in smaller transfer terms. The differences in the transfer terms of the loop and toe loop compared with the other jumps were probably due to the inclination of the leg at takeoff. In particular, in the Lutz and flip, where the transfer terms were almost zero, the toe pick legs were probably positioned almost directly beneath the COM at takeoff.

A large transfer term for angular momentum is generated by swinging the free leg, which has the second-highest mass after the trunk, around the COM. The angular momentum of the free leg continues to decrease while pivoting in the axel (7). In the present study, the angular momentum of the free leg in the axel was significantly smaller than that in the other jumps (Table 3). This suggests that the swung-up free leg was already drawn toward the COM before takeoff. The same may be true for the smaller transfer term of the free leg in the toe loop.

In addition, the transfer term of the free leg in the Lutz was significantly greater than that in the flip (Table 3). The Lutz and flip differ only on the side of the edge used for takeoff (Table 1). The Lutz takeoff involves the outer edge of the blade of the supporting (left) leg and is the only one of the six jump types in which the body undergoes a rotational movement in the opposite direction to the original direction of rotation in the air before takeoff. Thus, the Lutz likely generates a greater angular momentum of the free leg than the flip, which is a similar jumping method.

#### 4.2.4 Angular momentum patterns

Differences in the pattern of the proportion of the angular momentum of each body part relative to the angular momentum of the entire body were observed. In the Lutz and flip, which have similar jumping methods, the ratio of the free leg was the largest, followed by those of the trunk, right arm, left arm, and toe-pick leg (Figures 3D,E). The Salchow and loop showed a similar pattern, although there was a difference between the edge and toe jumps (Figures 3C,D). In both cases, the free leg, trunk, and takeoff or toe-pick leg followed to the same extent, followed by the left arm and then the right arm. In contrast, the axel showed a specific pattern compared with the other five jumps (Figure 3A). The proportion of angular momentum generated by each body part was similar for all body parts (16.9–23.2%). These differences in the angular momentum patterns of each body part suggest different strategies for generating angular momentum during the period from the approach to takeoff for the type of jump.

The findings on the angular momentum of the entire body and each body part obtained in this study may lead to improved athletic performance and the development of effective training methods. Participants in this study had high-level skating skills. Therefore, the results of this study may guide the performance of double-rotational jumps with sufficient success. For example, for athletes with low skating skills, it may be useful to practice jumping methods such that the pattern of angular momentum in each body part approaches the pattern shown in Figure 3, after generating a sufficiently large angular momentum in the approach.

### 4.3 Limitations and future directions

The present study only focused on angular momentum, and no other kinematic analyses were performed. Therefore, all relationships between the angular momentum and movement of the body discussed in this study are speculations based on previous studies and general findings in figure skating. The angular momentum of the entire body at takeoff was generated from the approach to the takeoff. Moreover, the moment of inertia should be regulated by the upper and lower limbs in the air to obtain the required number of rotations. Based on the results of this study, kinematic analyses should clarify how angular momentum is generated during the approach and how rotational velocity is acquired in the air.

Additionally, the number of rotations in the air is complexly related not only to the angular momentum of the entire body at takeoff but also to the vertical velocity, flight time, and moment of inertia. Therefore, simply increasing the angular momentum of the entire body at takeoff does not necessarily guarantee a successful triple- or quadruple-rotational jump. The results of the present study show that a sufficient amount of angular momentum is required for a successful double-rotational jump and that caution should be exercised in its application to jumps with a higher number of rotations.

## 5 Conclusion

In this study, the angular momentum at takeoff was compared for six different figure skating double jumps. The results showed that the angular momentum of the entire body was approximately the same, and a large local angular momentum term was generated in the trunk for all jumps. Although large angular momenta of the transfer terms were generated in the right and left arms and the free leg for all jumps, the patterns of them differed depending on the jump type. This suggests that the generation strategies of angular momenta may differ across the jump type. Skaters may improve their jump performance by training to move their arms and free legs in a manner that approximates the pattern of each jump. Additionally, the results also suggest that skaters may adjust their moment of inertia in the air, particularly in the axel and Salchow jumps, in order to achieve the necessary rotational velocity. These findings were obtained for the first time by comparing the six types of jumps. The angular momentum of high-level skaters will be an important indicator for future studies on treating lower-skilled skaters and focusing on triple and quadruple jumps. Based on these results, further analysis of the movements of body parts during the approach and of the moment of inertia in the air could clarify the mechanics of figure skating jumps.

## Data Availability

The raw data supporting the conclusions of this article will be made available by the authors, without undue reservation.

## References

[1] International Skating Union. ISU Communication 2475, Single & Pair Skating—Scale of Values Season 2022/23. Available online at: https://isu-d8g8b4b7ece7aphs.a03.azurefd.net/isudamcontainer/CMS/isucommunications/pdf/2475SPSOV20222317315 77372.pdf (Accessed March 20, 2025).

[2] Rauer T Pape HC Knobe M Pohlemann T Ganse B. Figure skating: increasing numbers of revolutions in jumps at the European and world championships. PLoS One. (2022) 17(11):e0265343. 10.1371/journal.pone.0265343 36449462 PMC9710745

[3] International Skating Union. Skating News, Ilia Malinin (USA) lands first quad Axel. Available online at: https://isu-skating.com/figure-skating/news/ilia-malinin-usa-lands-first-quad-axel/ (Accessed March 20, 2025).

[4] Sakurai S Ikegami Y Akiya I Asano K. Jump height in ladies single figure skating in the 18th winter Olympic games in Nagano 1998. ISBS Conference Proceedings Archive: Proceedings of the 17th International Symposium on Biomechanics in Sports (1999). p. 105–108

[5] King D Arnold A Smith S. A kinematic comparison of single, double, and triple axels. J Appl Biomech. (1994) 10:51–60. 10.1123/jab.10.1.51

[6] King D Smith S Higginson B Muncasy B Scheirman G. Characteristics of triple and quadruple toe-loops performed during the Salt Lake City 2002 Winter Olympics. Sports Biomech. (2004) 3(1):109–123. 10.1080/14763140408522833 15079991

[7] Albert W Miller D. Takeoff characteristics of single and double axel figure skating jump. J Appl Biomech. (1996) 12:72–87. 10.1123/jab.12.1.72

[8] Ridge ST McLean D Bruening D Richards J. Up in the air: the efficacy of weighted gloves in figure skating jumps. Sports Biomech. (2022) 23(12):2637–2648. 10.1080/14763141.2022.2046844 35306974

[9] Dapena J. Mechanics of rotation in the fosbury-flop. Med Sci Sports Exercise. (1980) 12:45–53. Available online at: https://journals.lww.com/acsm-msse/abstract/1980/21000/mechanics_of_rotation_in_the_fosbury_flop.10.aspx 7392902

[10] Ae M Tang H Yokoi T. Estimation of inertia properties of the body segments in Japanese athletes. J Soc Biomech Japan. (1992) 11:23–33. (In Japanese). 10.3951/biomechanisms.11.23

[11] Hinrichs RN. Upper extremity function in running. II: angular momentum considerations. J Appl Biomech. (1987) 3(3):242–263. 10.1123/ijsb.3.3.242

[12] Nagata Y Yoshida M. Toukeiteki Taju Hikakuho no Kiso (in Japanese) [Fundamentals of Statistical Multiple Comparison Methods]. Tokyo: Scientist Press Co (2015).

[13] Dapena J. A method to determine the angular momentum of a human body about three orthogonal axes passing through its center of gravity. J Biomech. (1978) 11:251–256. 10.1016/0021-9290(78)90051-9 711774

[14] Tang H. Evaluation of smash technique from the viewpoint of conservation of angular momentum. J Soc Biomech Japan. (1996) 13:33–40. (In Japanese). 10.5432/jjpehss.KJ00003391388

